# Improving Maternal Mental Health and Weight Control With a Mindfulness Blended Care Approach: Insights From a Randomized Controlled Trial

**DOI:** 10.2196/56230

**Published:** 2025-02-24

**Authors:** Kathrin Hassdenteufel, Mitho Müller, Harald Abele, Sara Yvonne Brucker, Johanna Graf, Stephan Zipfel, Armin Bauer, Peter Jakubowski, Jan Pauluschke-Fröhlich, Markus Wallwiener, Stephanie Wallwiener

**Affiliations:** 1 Department of Obstetrics and Gynecology University Hospital Heidelberg Heidelberg Germany; 2 Department of Psychology Ludwig-Maximilians-University Munich Germany; 3 Department of Women’s Health University Hospital Tübingen Tübingen Germany; 4 Department of Psychosomatic Medicine and Psychotherapy University Hospital Tübingenn Tübingen Germany; 5 German Centre for Mental Health (DZPG-Tuebingen) Tübingen Germany; 6 Department of Women’s Health Research Institute for Women’s Health University Hospital Tübingen Tübingen Germany; 7 Department of Gynecology Martin Luther University of Halle-Wittenberg Halle Germany; 8 Department of Obstetrics and Prenatal Medicine Martin Luther University of Halle-Wittenberg Halle Germany

**Keywords:** peripartum mental health, digital intervention, depression, anxiety, personal coaching, ehealth, pregnancy, maternal mental health, weight gain, mindfulness-based intervention, coaching, randomized controlled clinical trial, postpartum, treatment, electronic, effectiveness, women, digital intervention

## Abstract

**Background:**

Perinatal maternal mental health problems, such as depression and anxiety, are highly prevalent during pregnancy and post partum. Electronic mindfulness-based interventions (eMBIs) are a promising treatment option, which can be provided in a low-threshold, cost-effective manner. However, research underscores the fact that face-to-face coaching sessions are more effective than solely digital methods. A blended care approach (eMBI with direct face-to-face coaching) could amplify the therapeutic impact on maternal mental health and weight gain during the perinatal period.

**Objective:**

We investigated whether combining an eMBI intervention with face-to-face personal support significantly improves maternal mental health, and whether the intervention can influence weight gain in affected women during pregnancy.

**Methods:**

A community-based sample of 460 pregnant women with a singleton pregnancy who screened positive for depression was enrolled in a multicenter randomized controlled trial (RCT) including the University Hospitals of Heidelberg and Tübingen as well as more than 200 gynecological practices within the state of Baden-Württemberg in Germany between February 2019 and October 2020. Participating women were randomized 1:1 to the control group (CG) or intervention group (IG) that received access to an 8-week pregnancy-adapted eMBI between the 29th and 36th gestational week. In a subanalysis, we grouped participants in those receiving only the initial face-to-face coaching session at recruitment (no personal coaching) and those with ≥2 personal coaching sessions. Primary outcome measures were severity of depressive symptoms using the Edinburgh Postnatal Depression Scale, anxiety using the State-Trait Anxiety Inventory, the Pregnancy-Related Anxiety Questionnaire, the Freiburg Mindfulness Inventory, and the Patient Health Questionnaire; secondary outcome measure, BMI.

**Results:**

In the final sample, 137 CG women and 102 IG women received only one coaching session, whereas 37 CG women and 40 IG women received at least 2 (mean 2.3, SD 0.7) coaching sessions. The analyses were adjusted for significant confounders. The IG’s mindfulness scores increased significantly (*F*_1.873,344.619_=4.560, *P*=.01, η²=0.024, ω²=0.012) regardless of coaching frequency. Both general anxiety (*F*_12,129_=2.361, *P*=.01, η²=0.0180, ω²=0.100) and depression symptoms (*F*_4.758, 699.423_=3.033, *P*=.01, η²=0.020, ω²=0.009) were significantly lower in the group that received ≥2 coaching sessions than in the no-personal-coaching group. In the group receiving ≥2 coaching sessions, BMI generally was lower in the IG than in the CG (*F*_3.555,444.416_=4.732, *P*=.002, η²=0.036, ω²=0.013).

**Conclusions:**

Adding a minimal amount of PC to the digital eMBI increased mindfulness and decreased birth-related anxiety, symptoms of depression, and anxiety in at-risk pregnant women. Favorable effects on gestational weight gain were found in the respective IGs, the strongest effect being within the PC group. This blended digital health approach amplifies the effectiveness of the digital intervention.

**Trial Registration:**

German Clinical Trials Register DRKS00017210; https://www.drks.de/search/de/trial/DRKS00017210

## Introduction

During the perinatal period, pregnant women face an elevated risk of developing anxiety and depressive disorders. While prenatal depression affects 10%-25% of pregnant women [1], up to one-third experience depression and anxiety disorders after delivery [2,3].

The etiology of these disorders is multifactorial, ranging from obstetrical, high-risk pregnancies and psychological distress to the manifestation of previous depression or anxiety disorders [4]. Once present, perinatal mental disorders can, in turn, lead to pregnancy complications, sleep disturbances, suicidal tendencies, high-risk behavior, and overall poor maternal health-related quality of life [5-7]. Moreover, maternal anxiety and depression increase the risk of preterm birth and newborn low birth weight and have a negative impact on infant behavioral, motor, and cognitive development [8,9].

Psychological distress, in turn, is associated with greater gestational weight gain (GWG) during pregnancy and a higher risk for impaired insulin resistance, potentially resulting in adverse outcomes for both mother and child [10]. As women are at higher risk for gestational diabetes, cesarean section, large-for-gestational-age fetal growth, and postpartum weight retention, prevention at this stage is crucial [11]. As excessive GWG can be found in approximately 50% of all pregnancies, thus presenting a major global health burden, prenatal lifestyle interventions and prevention measures are warranted [12].

Meanwhile, digital interventions such as mindfulness approaches have proven to be helpful in supporting pregnant women with mental health symptoms and in preventing excessive weight gain [13]. We recently demonstrated the beneficial effects of an electronic mindfulness-based intervention (eMBI) in a randomized controlled clinical trial (RCT) [14] with an 8-week digitally guided intervention. The intervention significantly increased maternal mindfulness and significantly decreased birth-related anxiety. However, while the intervention significantly impacted the prevalence of postpartum depressiveness, symptoms of depression and general anxiety were not significantly reduced during the intervention. Comparable outcomes in diminishing maternal distress have been highlighted in previous research as well [15]. Lönneberg et al [16] conducted an RCT to evaluate the effects of a face-to-face mindfulness program (Mindfulness-Based Childbirth and Parenting Program) on stress, depression, and mood of pregnant women. Integrating perinatal health insights with mindfulness exercises, the program yielded promising results: participants reported fewer depressive symptoms, diminished stress levels, an increased sense of mindfulness, and a positive state of mind after completing the program.

However, this literature is sparse regarding the prevention of glucose intolerance. Research has shown favorable effects of a personal mindfulness intervention in improving perinatal stress and maternal weight gain, though without reaching significance [10].

While digital interventions have proven useful, we questioned whether their effectiveness could be optimized by combining eMBI with a minimum of personal support, such as face-to-face interventions, in a blended care model. Digital health care interventions have generally shown high acceptability rates and represent far-reaching, relatively cost-effective methods to support health care on a low-threshold basis [17]. However, despite the considerable growth of digital interventions in the last decade, previous research has shown that such interventions tend to lack sensitivity and may not adequately meet individual needs [18]. Therefore, flexible solutions are warranted, and ongoing studies are increasingly focusing on blended digital health solutions with human support in order to enhance patient engagement and satisfaction [19]. Favorable results in combining conventional digital tools with a human touch, which has been identified as a key component, were demonstrated in an RCT focusing on the promotion of healthy GWG [20]. In line with this, Chiauzzi and Newell [19] assert that flexible use of apps based on a tailored strategy is more appropriate than reliance on the app alone. Therefore, adding personal support to digital mindfulness programs may increase their positive outcome. As digital-only methods frequently underperform compared to face-to-face approaches in addressing depression, general anxiety, and GWG, adding a personal touch seems imperative.

Personal coaching (PC) through smartphones or video may improve the mental health of pregnant women, fostering deeper interactions, on the one hand, and facilitating health-focused conversations with health care personnel, on the other. Building on this premise, we aimed to extend previous research by comparing 2 different clinical approaches, that is, eMBI and PC. Expanding upon our previous RCT, we investigated the combined efficacy of personal support and coaching with an electronic mindfulness program in this study, specifically examining its effects on depression and anxiety as well as on maternal weight gain as a secondary outcome in pregnant women.

## Methods

### Patients

Participating centers were the university hospitals of Heidelberg and Tübingen, along with over 200 gynecological practices in the state of Baden-Wuerttemberg, Germany, as detailed in our previous publication [14]. Briefly, we screened 5299 pregnant women using the Edinburgh Postnatal Depression Scale (EPDS) between February 2019 and October 2020. Those scoring above 9 were enlisted to participate in an 8-week eMBI from their 29th to 36th gestational weeks. Demographic and psychometric data were collected before, during, and up to 6 months after the intervention and compared with the control group (CG).

For this subanalysis, we compared participants who received more than one face-to-face coaching session during the study period (personal coaching group [PC group]), with participants who received only the first mandatory session (no personal coaching [NPC group]). The effect of PC on psychometric parameters was evaluated in the intervention groups (IG) and CGs. The clinical study phase was conducted over a 2-year period between January 1, 2019, and December 31, 2020. The individual study period was 13 months.

### Ethical Considerations

The study was approved by the ethics committees of the Medical Faculties of the Universities of Heidelberg (S-744/2018) and Tübingen (952/2018BO2) and was conducted in accordance with the ethical standards of the Declaration of Helsinki. All personal data were collected in accordance with the principles of confidentiality and the European General Data Protection Regulation (EU-GDPR). All participants provided a signed written consent and received €100 (roughly US $102) each as financial compensation. The study participants had the right to request information about the personal data collected from them. Participation in the study was voluntary after all study content and objectives had been declared and written consent had been given and could be withdrawn at any time without any disadvantages for the patient. Patient data and clinical data are recorded pseudonymously and stored centrally in the eCRF. This study follows the CONSORT (Consolidated Standards of Reporting Trials) statement (Multimedia Appendix 1) and the SPIRIT (Standard Protocol Items: Recommendations for Intervention Trials) guidelines [21]. The study was registered with the German Clinical Trials Registry (DRKS00017210).

### Interventions

Expectant mothers were randomized 1:1 to the IG, where they were granted access to eMBI (IG=eMBI), or the CG, which received standard care, including an initial psychological session (CG=treatment as usual [TAU]). The eMBI, starting at 29 weeks gestation, consisted of eight weekly 45-minute sessions. These sessions provided psychoeducational and obstetrical content, along with pregnancy-adapted mindfulness exercises. Both groups completed digital questionnaires on a regular basis: every 2 weeks during the intervention (T1-T5) period and at 1 and 5 months post partum (T6 and T7). Before randomization, all women participated in a mandatory psychological assessment. Enhanced personalized care encompassed the telephone availability of supervised and professionally trained providers and access to psychological support when needed.

The eMBI was developed by a multidisciplinary team that included gynecologists, psychologists, and midwives. The questionnaires assessed sociodemographic and medical data, physiological measures, and self-reported data on maternal mental health. In addition, each participating woman had weekly access to a self-developed digital pregnancy guide with educational and validated medical content focused on pregnancy and childbirth. The modules included for example the following topics: fears and worries about birth or parenting, coping with stress, birth-related pain control, and outlook puerperium.

### Primary Outcome Measures

#### The EPDS

The EPDS, originally developed in 1998 by Cox et al [22] and translated into German by Bergant et al [23], is a 10-item self-rating scale that assesses depressive symptoms in the peripartum. The EPDS has proven to be a valid and sensitive instrument for predicting depressive disorders not only in the postnatal period but also prenatally [24]. The most commonly used cut-off score of 9 (EPDS>9) has shown a sensitivity of 0.96 and a specificity of 1.00 [25].

#### State-Trait Anxiety Inventory

Perinatal anxiety was measured using the German version of the State-Trait Anxiety Inventory (STAI) during the perinatal period. The questionnaire consists of two scales: The STAI-S (state scale) assesses anxiety as a temporary condition, encompassing feelings of tension, nervousness, and worry, whereas the STAI-T (trait scale) refers to dispositional anxiety over time. The sum, ranging from 20 to 80, indicates the level of situational and dispositional anxiety [26].

#### Pregnancy-Related Anxiety Questionnaire–Revised

The Pregnancy-Related Anxiety Questionnaire–Revised (PRAQ-R) is an abbreviated 10-item self-report measure of pregnancy and childbirth anxiety and is a valid predictor of birth and childhood outcomes [27-29]. Items include fear of childbirth, concerns about bearing a physically or mentally handicapped child, and concerns about one’s appearance [30].

### Secondary Outcomes

#### Freiburg Mindfulness Inventory

The Freiburg Mindfulness Inventory (FMI-14, also known as FFA-14) was developed according to the Buddhist rules of life. It defines mindfulness as the tendency to act in a mindful way, measured as a personal characteristic [31,32]. We used the abbreviated German version consisting of 14 Likert-scale (1-4) items [33].

#### Patient Health Questionnaire

The Patient Health Questionnaire (PHQ-D), tailored for the practical screening of mental disorders in primary care, directly measures the diagnostic criteria of the *DSM-IV* (*Diagnostic and Statistical Manual of Mental Disorders* [Fourth Edition]) [34]. It has been shown to be a valid and well-accepted self-rating instrument for use in research and clinical practice, also in its German rendition [35]. The PHQ-D measures the following scales with 78 items: somatoform, depressive, anxiety, eating disorders, and alcohol abuse. It also includes items on psychosocial functioning, stress experiences, and critical life events.

#### BMI

Participants responded to items about body height and weight. Weight was assessed at all time points. In addition, at T1, weight before pregnancy was assessed retrospectively (T0). Thus, we were able to compute the BMI for 8 time points according to the respective formula:



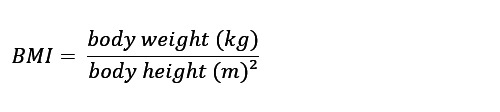



#### Statistical Analysis Plan

All analyses were conducted using SPSS Statistics for Windows (version 29.0.0.0-241; IBM Corp). Little’s Missing-completely-at-random test was used to ensure that missing data due to dropouts and missing values were valid for our analyses [36] Moreover, the groups were tested for comparability regarding sociodemographic and medical third variables by means of *t* tests, Mann-Whitney *U* tests and chi-square tests. If differences were significant, the respective variables were analyzed for associations with the outcome variables and included as covariates.

The manipulation check (analyses regarding mindfulness as indexed by the FFA-14 scores) as well as the primary and secondary analyses were conducted using (multivariate) ANOVA with repeated measurements corrected for significant confounders. Mauchly’s sphericity test was used to determine whether the sphericity assumption had been violated. If significant, repeated measures *df*s were corrected using Huynh–Feldt or Greenhouse-Geisser correction, depending on the degree of violation. Due to the exploratory nature of the current analyses, the critical, local α-errors were not adjusted and set to a conventional level of α*_local_*=.05. Partial η² and ω² are used as effect sizes. These are sample-based and population-based estimators of explained variances, respectively. According to Cohen [37], η²/ω²=0.01 represents small, η²/ω²=0.06 represents medium, and η²/ω²=0.14 represents large effects. Dunn’s multiple comparison procedure [38] was performed as a post hoc test for significant effects relevant to the hypotheses. This procedure results in a minimum significant difference (ψ).

The final sample consisted of 460 participants. However, due to dropouts and further missing values, the number of valid cases varies. Post hoc power calculations (calculated with G*Power; version 3.1.9.7) [39,40] for nonsignificant results are reported in the results section. However, in our ANOVAs, mainly, the power was virtually 1–β=1.0 for large (*f*=0.40) within- and between-subject effects as well as for medium-sized within-subject effects (*f*=0.25).

Main and interaction effects are not reported or interpreted in detail if the respective factors are significantly interacting with additional factors. Also, the main effects of confounders are not reported and interpreted in detail.

## Results

### Descriptive Characteristics

**Figure 1:**
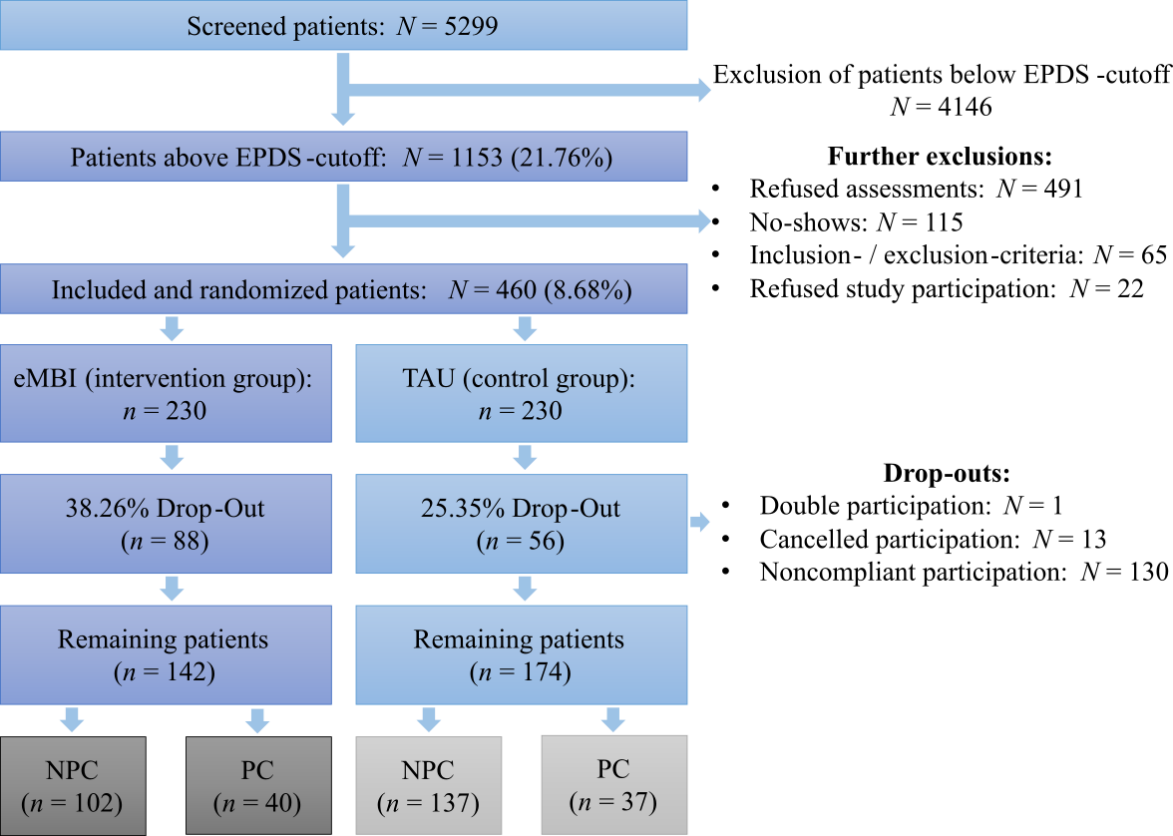
Participant flowchart. EPDS: Edinburgh Postnatal Depression Scale; eMBI: electronic mindfulness-based intervention; TAU: treatment as usual; NPC: no personal coaching; PC: personal coaching.

The flow chart is presented in Figure 1. Results of the Missing-completely-at-random test were not significant (*χ*²_25,073_=25,104.7, *P*=.44). All sociodemographic characteristics and tests on comparability are demonstrated in Tables 1 and 2. The IG and the CG statistically significantly differed regarding the PHQ score, the number of children at home, and the educational level (refer to Table 1): the IG scored lower in the PHQ stress evaluation, had more children at home, and had a lower educational level than the CG. There were no significant differences between the NPC and the PC groups (refer to Table 2).

The potential confounders identified were correlated with the outcome variables: The PHQ stress evaluation was significantly associated with every outcome at every measurement point, except the BMI. The associations (*r*) ranged between 0.201 and 0.479 (*P*<.001), showing the highest association with the STAI-T at T6 and the lowest association with the PRAQ-R at T5. The numbers of children at home were significantly associated with the FFA-14, the STAI-S, and STAI-T, as well as the PRAQ-R (ρ ranging between–0.241 and 0.173) for the highest positive association with the STAI-S at T1 and the highest negative association with the PRAQ-R at T1 (levels of significance [*P*] ranging between <.001 and .048). The level of education was significantly associated with the EPDS, STAI-S, and STAI-T, and the BMI (ρ ranging between–0.346 and –0.122) for the highest negative association with the BMI at T4 and the lowest negative association with the EPDS at T1 (levels of significance [*P*] ranging between <.001 and .04). Consequently, we controlled these variables as covariates in the respective analyses. Descriptive statistics of outcome measures are shown in Table 3.

**Table 1:** Demographics and tests on comparability of the subgroups TAUM^a^ versus eMBI^b^.

Characteristics			General		TAU		eMBI		Statistics^c^			*P* value	
Maternal age (years), mean (SD)			32.6 (4.3)		32.8 (4.6)		32.3 (4.7)		0.95 (314)^d^			.35	
Gestation age at study inclusion (weeks), mean (SD)			21.2 (4.3)		21.4 (4.2)		21.0 (4.3)		0.78 (307)^d^			.43	
Gestation age at birth (weeks), mean (SD)			39.2 (1.7)		39.2 (1.7)		39.2 (1.6)		0.14 (290)^d^			.89	
Infant body weight (grams), mean (SD)			3,370.4 (475.9)		3,411.8 (438.6)		3,319.4 (515.3)		1.70 (306)^d^			.09	
Infant body length (cm), mean (SD)			51.5 (2.6)		51.7 (2.5)		51.2 (2.6)		1.52 (300)^d^			.13	
PHQ^e^ stress evaluation (points), mean (SD)			6.8 (3.4)		7.2 (3.7)		6.3 (3.0)		2.28 (313)^d^			.02	
**Maternal education frequencies (%)**										8396.5^f^			<.01
	University entrance qualification	149 (51.4)		97 (58.1)		52 (42.3)							
	University of applied sciences entrance qualification	48 (16.6)		29 (17.4)		19 (15.4)							
	High secondary qualification	78 (26.9)		32 (19.2)		46 (37.4)							
	Low secondary qualification	14 (4.8)		8 (4.8)		6 (4.9)							
	No school leaving qualification	1 (0.3)		1 (0.6)		0 (0.0)							
**Level of maternal occupation frequencies (%)**										9150.0^f^			.84
	Prohibition notice	113 (40.9)		68 (42.5)		45 (38.8)							
	Unemployed	35 (12.7)		16 (10.0)		19 (16.4)							
	Part-time employed	60 (21.7)		33 (20.6)		27 (23.3)							
	Full-time employed	68 (24.6)		43 (26.9)		25 (21.6)							
**Household net income frequencies (%)**										8744.5^f^			.15
	< 1.500 €	70 (24.8)		36 (21.8)		34 (29.1)							
	1.500-2.999 €	128 (45.4)		76 (46.1)		52 (44.4)							
	3.000-4.999 €	62 (22.0)		39 (23.6)		23 (19.7)							
	5.000-8.000 €	21 (7.4)		13 (7.9)		8 (6.8)							
	> 8.000 €	1 (0.4)		1 (0.6)		0 (0.0)							
**Civil status frequencies (%)**										5.35 (5)^g^			.34
	Married	192 (65.8)		113 (67.3)		79 (63.7)							
	Partnership	94 (32.2)		53 (31.5)		41 (33.1)							
	Single	5 (1.7)		1 (0.6)		4 (3.2)							
	Divorced	1 (0.3)		1 (0.6)		0 (0.0)							
**Country of origin frequencies (%)**										19.17 (17)^g^			.17
	Germany	249 (85.3)		140 (83.3)		109 (87.9)							
	Other	43 (14.7)		28 (16.7)		15 (12.1)							
**Current psychotherapy frequencies (%)**										0.01 (1)^g^			.99
	False	55 (17.5)		30 (17.3)		25 (17.7)							
	True	259 (82.5)		143 (82.7)		116 (82.3)							
**Gravidity frequencies (%)**										11,822.0^f^			.48
	1st pregnancy	143 (45.3)		82 (47.1)		61 (43.0)							
	2nd pregnancy	85 (26.9)		44 (25.3)		41 (28.9)							
	3rd pregnancy	47 (14.9)		29 (16.7)		18 (12.7)							
	≥4th pregnancy	41 (13.0)		19 (10.9)		22 (15.5)							
**Parity frequencies (%)**										11,675.5^f^			.35
	1st birth	177 (56.0)		102 (58.6)		75 (52.8)							
	2nd birth	103 (32.6)		53 (30.5)		50 (35.2)							
	3rd birth	30 (9.5)		15 (8.6)		15 (10.6)							
	≥4th birth	6 (1.9)		4 (2.3)		2 (1.4)							
**Number of children at home frequencies (%)**										9075.5^f^			<.05
	No child	167 (57.4)		105 (62.5)		62 (50.4)							
	One child	96 (33.0)		49 (29.2)		47 (38.2)							
	Two children	25 (8.6)		12 (7.1)		13 (10.6)							
	Three or more children	3 (1.0)		2 (1.2)		1 (0.8)							
**Birth mode frequencies (%)**										2.29 (3)^g^			.51
	Spontaneous	179 (57.9)		103 (60.6)		76 (54.7)							
	Primary c-section	42 (13.6)		23 (13.5)		19 (13.7)							
	Secondary c-section	64 (20.7)		30 (17.6)		34 (24.5)							
	Vaginal operative	24 (7.8)		14 (8.2)		10 (7.2)							
**Infant sex frequencies (%)**										0.34 (1)^g^			.64
	Female infants	126 (40.6)		72 (42.1)		54 (38.8)							
	Male infants	184 (59.4)		99 (57.9)		85 (61.2)							
**Infant APGAR values after 10 min. frequencies (%)**										11,053.0^f^			.69
	10	279 (92.7)		154 (92.2)		125 (93.3)							
	9	16 (5.3)		8 (4.8)		8 (6.0)							
	8	5 (1.7)		4 (2.4)		1 (0.7)							
	7	1 (0.3)		1 (0.6)		0 (0.0)							

^a^TAU: treatment as usual.

^b^eMBI: electronic mindfulness-based intervention.

^c^Empirical α-error.

^d^*t* test (df) values.

^e^PHQ: Patient Health Questionnaire.

^f^Mann-Whitney *U* tests.

^g^Chi-square (df) values.

**Table 2:** Demographics and tests on comparability of the subgroups NPC^a^ versus PC^b^.

Characteristics		General	NPC	PC	Statistics^c^		*P* value	
Maternal age (years), mean (SD)		32.6 (4.3)	32.4 (4.5)	32.9 (4.9)	0.73 (314)^d^		.47	
Gestation age at study inclusion (weeks), mean (SD)		21.2 (4.3)	21.5 (4.3)	20.6 (4.3)	1.59 (307)		.11	
Gestation age at birth (weeks), mean (SD)		39.2 (1.7)	39.3 (1.5)	39.0 (2.2)	1.57 (290)		.12	
Infant body weight (grams), mean (SD)		3370.4 (475.9)	3363.8 (477.6)	3390.9 (473.4)	0.43 (306)		.67	
Infant body length (cm), mean (SD)		51.5 (2.6)	51.3 (2.6)	52.0 (2.4)	1.80 (300)		.07	
PHQ^e^ stress evaluation (points), mean (SD)		6.8 (3.4)	6.7 (3.4)	7.0 (3.5)	0.56 (313)		.58	
**Maternal education frequencies (%)**						7119.5^f^		.37
	University entrance qualification	149 (51.4)	111 (50.2)	38 (55.1)				
	University of applied sciences entrance qualification	48 (16.6)	37 (16.7)	11 (15.9)				
	High secondary qualification	78 (26.9)	59 (26.7)	19 (27.5)				
	Low secondary qualification	14 (4.8)	13 (5.9)	1 (1.4)				
	No school leaving qualification	1 (0.3)	1 (0.5)	0 (0.0)				
**Household net income frequencies (%)**						7096.5^f^		.85
	< 1.500 €	70 (24.8)	54 (25.1)	16 (23.9)				
	1.500 - 2.999 €	128 (45.4)	94 (43.7)	34 (50.7)				
	3.000 - 4.999 €	62 (22.0)	53 (24.7)	9 (13.4)				
	5.000 - 8.000 €	21 (7.4)	13 (6.0)	8 (11.9)				
	> 8.000 €	1 (0.4)	1 (0.5)	0 (0.0)				
**Level of maternal occupation frequencies (%)**						7013.0^f^		.91
	Prohibition notice	113 (40.9)	86 (41.3)	27 (39.7)				
	Unemployed	35 (12.7)	24 (11.5)	11 (16.2)				
	Part-time employed	60 (21.7)	46 (22.1)	14 (20.6)				
	Full-time employed	68 (24.6)	52 (25.0)	16 (23.5)				
**Civil status frequencies (%)**						4.23 (5)^g^		.50
	Married	192 (65.8)	143 (64.4)	49 (70.0)				
	Partnership	94 (32.2)	75 (33.8)	19 (27.1)				
	Single	5 (1.7)	3 (1.4)	2 (2.86)				
	Divorced	1 (0.3)	1 (0.5)	0 (0.0)				
**Country of origin frequencies (%)**						17.24 (17)^g^		.32
	Germany	249 (85.3)	192 (86.5)	57 (81.4)				
	Other	43 (14.7)	30 (13.5)	13 (18.6)				
**Current psychotherapy frequencies (%)**						0.27 (1)^g^		.60
	False	55 (17.5)	40 (16.9)	15 (19.5)				
	True	259 (82.5)	197 (83.1)	62 (80.5)				
**Gravidity frequencies (%)**						8127.0^f^		.10
	1st pregnancy	143 (45.3)	114 (47.7)	29 (37.7)				
	2nd pregnancy	85 (26.9)	64 (26.8)	21 (27.3)				
	3rd pregnancy	47 (14.9)	31 (13.0)	16 (20.8)				
	≥ 4th pregnancy	41 (13.0)	30 (12.6)	11 (14.3)				
**Parity frequencies (%)**						2964.0^f^		.89
	1st birth	177 (56.0)	139 (58.2)	38 (49.4)				
	2nd birth	103 (32.6)	74 (31.0)	29 (37.7)				
	3rd birth	30 (9.5)	22 (9.2)	8 (10.4)				
	≥ 4th birth	6 (1.9)	4 (1.7)	2 (2.6)				
**Number of children at home frequencies (%)**						7611.0^f^		.82
	No child	167 (57.4)	129 (58.4)	38 (54.3)				
	One child	96 (33.0)	68 (30.8)	28 (40.0)				
	Two children	25 (8.6)	22 (10.0)	3 (4.3)				
	Three or more children	3 (1.0)	2 (0.9)	1 (1.4)				
**Birth mode frequencies (%)**						1.86 (3)^g^		.60
	Spontaneous	179 (57.9)	135 (57.7)	44 (58.7)				
	Primary c-section	42 (13.6)	35 (15.0)	7 (9.3)				
	Secondary c-section	64 (20.7)	46 (19.7)	18 (24.0)				
	Vaginal operative	24 (7.8)	18 (7.7)	6 (8.0)				
**Infant sex frequencies (%)**						0.45 (1)^g^		.59
	Female infants	126 (40.6)	98 (41.7)	28 (37.3)				
	Male infants	184 (59.4)	137 (58.3)	47 (62.7)				
**Infant APGAR** ^h^ **values after 10-min frequencies (%)**						8058.0^f^		.37
	10	279 (92.7)	213 (93.4)	66 (90.4)				
	9	16 (5.3)	12 (5.3)	4 (5.5)				
	8	5 (1.7)	3 (1.3)	2 (2.7)				
	7	1 (0.3)	0 (0.0)	1 (1.4)				

^a^NPC: no personal coaching.

^b^PC: personal coaching.

^c^Empirical α-error.

^d^*t* test (df) values.

^e^PHQ: Patient Health Questionnaire.

^f^Mann-Whitney *U* test.

^g^Chi-square (df) value.

^h^APGAR: Appearance, Pulse, Grimace, Activity and Respiration.

**Table 3:** Descriptive statistics of outcome measures.

Time point		Sample size (N)	Minimum	Maximum	Mean (SD)	SE
**FFA-14^a^**						
	T1	285	15.00	54.00	33.50 (6.95)	0.41
	T5	287	15.00	53.00	35.01 (6.77)	0.40
	T7	228	17.00	55.00	35.57 (7.80)	0.52
**Edinburgh Postnatal Depression Scale**						
	T1	291	1.00	26.00	11.36 (4.98)	0.29
	T2	294	0.00	24.00	10.90 (4.93)	0.29
	T3	302	0.00	25.00	10.35 (4.92)	0.28
	T4	297	0.00	26.00	10.14 (5.23)	0.30
	T5	294	0.00	26.00	9.99 (5.66)	0.33
	T6	217	0.00	28.00	9.12 (5.94)	0.40
	T7	247	0.00	26.00	8.87 (5.48)	0.35
**State-Trait Anxiety Inventory-state scale**						
	T1	290	22.00	75.00	48.59 (11.04)	0.65
	T2	293	22.00	79.00	48.42 (11.12)	0.65
	T3	301	20.00	80.00	47.17 (11.67)	0.67
	T4	297	22.00	77.00	46.88 (12.16)	0.71
	T5	293	21.00	79.00	47.19 (12.21)	0.71
	T6	217	20.00	80.00	42.12 (12.77)	0.87
	T7	244	20.00	79.00	42.40 (12.47)	0.80
**State-Trait Anxiety Inventory-trait scale**						
	T1	289	21.00	79.00	48.83 (10.69)	0.63
	T2	293	22.00	73.00	48.23 (10.84)	0.63
	T3	301	21.00	75.00	46.82 (11.15)	0.64
	T4	297	22.00	78.00	45.77 (11.33)	0.66
	T5	293	22.00	76.00	45.91 (11.66)	0.68
	T6	214	20.00	78.00	42.50 (12.89)	0.88
	T7	242	20.00	75.00	42.91 (12.52)	0.81
**Pregnancy-Related Anxiety Questionnaire**						
	T1	288	10.00	50.00	25.76 (7.54)	0.44
	T2	293	12.00	48.00	25.89 (7.61)	0.44
	T3	301	10.00	50.00	25.50 (7.94)	0.46
	T4	297	10.00	47.00	25.36 (7.80)	0.45
	T5	294	10.00	50.00	25.55 (8.46)	0.49
**BMI**						
	T0	456	13.36	59.18	24.70 (5.58)	0.26
	T1	455	14.67	48.67	26.42 (5.25)	0.25
	T2	362	15.70	47.62	28.09 (5.12)	0.27
	T3	362	16.04	48.67	28.48 (4.99)	0.26
	T4	354	16.37	49.37	28.82 (5.07)	0.27
	T5	337	16.71	50.77	29.06 (5.11)	0.28
	T6	209	17.63	41.51	26.15 (5.06)	0.35
	T7	247	14.70	49.72	25.47 (5.39)	0.34

^a^FFA-14: Freiburg Mindfulness Inventory.

### Manipulation Check (FFA-14)

As covariates a 2 (group) × 2 (coaching) × 3 (time) ANOVA with PHQ stress evaluation and the number of children at home were used. The assumption on sphericity was significantly violated (*P*<.001) and thus Huynh-Feldt correction was applied (ε=.936). There was a significant main effect of time (*P*<.001).

**Figure 2:**
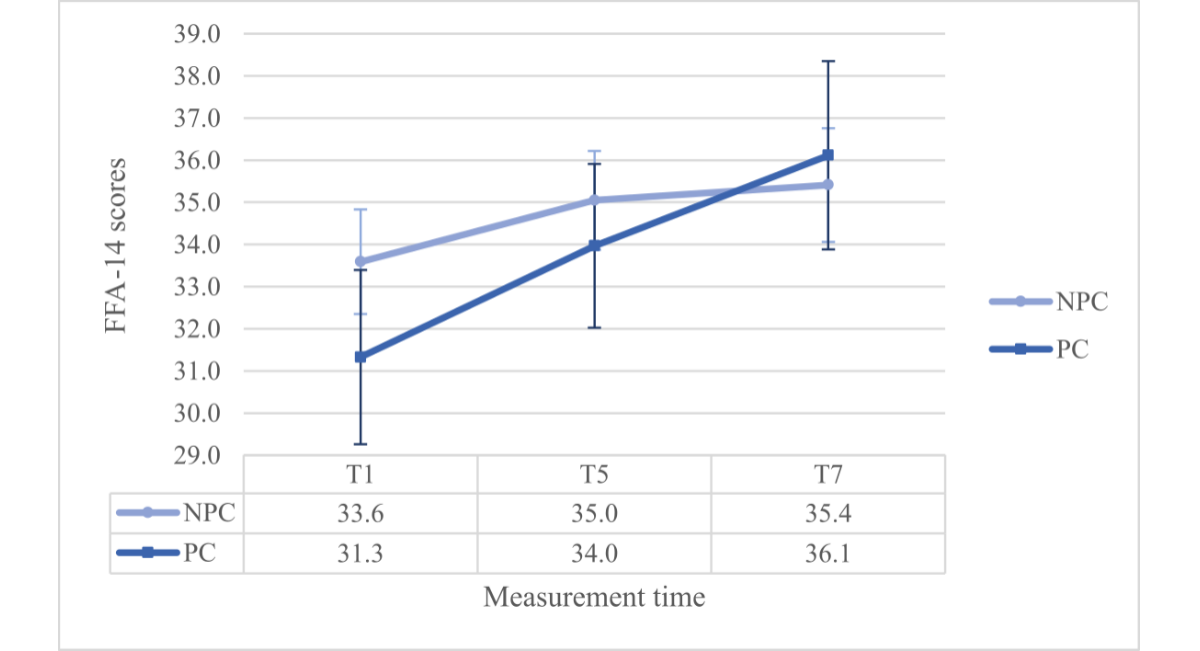
Interaction effect between coaching and time regarding FFA-14 scores. GW: gestational week; T1: 28th GW; T5=36th GW; T7=5 months postpartum; NPC: no personal coaching; PC: personal coaching.

Moreover, a significant interaction effect was observed between group and time (*F*_1.873,344.619_=4.560, *P*=.01, η²=0.024, ω²=0.012). Dunn’s post hoc test (Ψ_Dunn_=1.9) revealed a significant increase from T1 to T5 and T7 for the IG, but also an increase from T1 and T5 to T7 for the CG. However, the IG score is significantly higher than the CG score at T5. In addition, there was a significant interaction effect between coaching and time (*F*_1.873,344.619_=4.585, *P*=.01, η²=0.024, ω²=0.012). Dunn’s post hoc test (Ψ_Dunn_=2.4) revealed a significant increase from T1 to T5 and T7 but only for the PC group (refer to Figure 2).

Furthermore, a significant main effect of the PHQ stress evaluation was observed. There were no other significant main (*P*≥.14) or interaction effects (*P*≥.09).

We calculated a power of 1–β=.927 for medium-sized between-subject effects and 1–β=.816 for small within-subject effects (*f*=0.10). Only small between-subject effects cannot be ruled out sufficiently with 1–β=0.232.

### Symptoms of Depression According to EPDS

As covariates a 2 (group) × 2 (coaching) × 7 (time) ANOVA with PHQ stress evaluations and the highest school educational level were used. The assumption on sphericity was significantly violated (*P*<.001) and thus Huynh-Feldt correction was applied (ε=.793).

We found significant main effects of the PHQ stress evaluation (*P*<.001) and maternal education (*P*=.01). Moreover, the main effect of the group was significant (*F*_1,147_=7.845, *P*=.006, η²=0.051, ω²=0.04), revealing a generally lower EPDS score for the IG (mean 9.3, SE 0.5) than for the CG (mean 11.2, SE 0.4).

**Figure 3:**
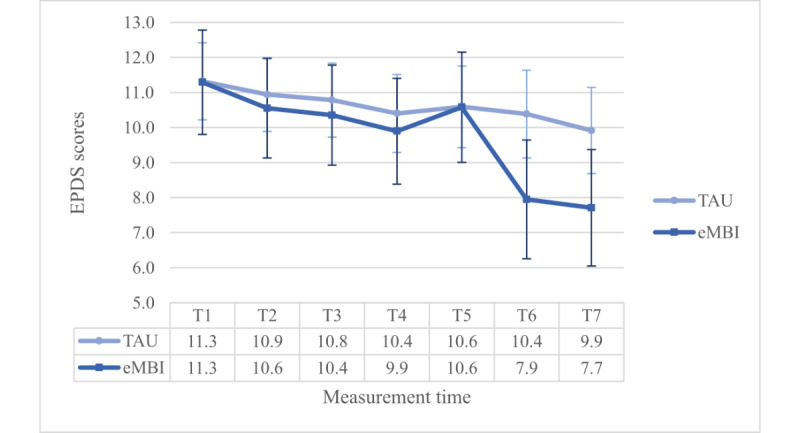
Group x time—interaction effect regarding EPDS scores for the NPC group. eMBI: electronic mindfulness-based intervention; GW: gestational week; PP: postpartum; T1=28th GW; T2=30th GW; T3=32nd GW; T4=34th GW, T5=36th GW, T6=1 month, T7 = 5 months PP, TAU: treatment as usual.

**Figure 4:**
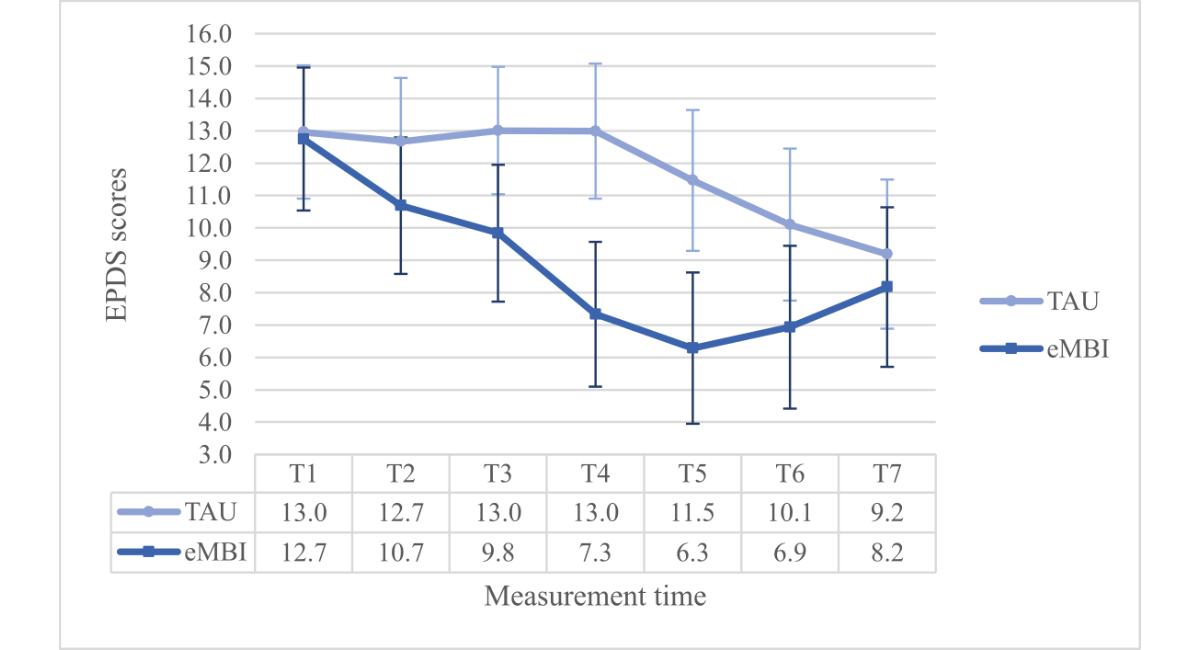
Group x time-interaction effect regarding EPDS scores for the PC group. T1 = 28th gestational week (GW), T2 = 30th GW, T3 = 32nd GW, T4 = 34th GW, T5 = 36th GW, T6 = 1 month postpartum (pp), and T7 = 5 months pp, TAU: treatment as usual, eMBI: electronic mindfulness-based intervention.

The coaching time—interaction effect was significant, too (*P*=.04). Most importantly, this interaction term was enriched by a significant 3-way interaction term (*F*_4.758,699.423_=3.033, *P*=.01, η²=0.020, ω²=0.009): Dunn’s post hoc test (Ψ_Dunn_=4.1) revealed significant differences at T4 and T5 between the IG and the CG, but only in the PC group (Figures 3 and 4).

Findings were nonsignificant for all other between- (*P*≥.12) and within-subject effects (*P*≥.07). The power was 1–β=0.983 for medium-sized (*f*=0.40) between-subject effects. Only small within-subject and between-subject effects (*f*=0.10) cannot be ruled out sufficiently with 1–β=.590 and 1–β=.313.

### General Anxiety According to the STAI

As covariates a 2 (group) 2 (coaching) 7 (time) MANOVA with PHQ stress evaluations, the number of children at home, and the highest educational level were used. There was a significant main effect of PHQ stress evaluation (*P*<.001). Moreover, a significant main effect of group was observed (*P*=.04), which was enriched by a significant 2-way interaction term between group and time (*P*=.01) as well as a significant 3-way interaction term (*F*_12,129_=2.361, *P*=.01, η²=0.0180, ω²=0.100).

Findings were nonsignificant for all other between- (*P*≥.29) and within-subject effects (*P*≥.23). The power was 1–β=.920 for medium-sized (*f*=0.40) within- and .981 for medium-sized between-subject effects. Only small within-subject and between-subject effects (*f*=0.10) cannot be ruled out sufficiently with 1–β=0.175 and 1–β=0.307. Therefore, we calculated two 2 (group) 2 (coaching) 7 (time) post hoc ANOVAs (one for each STAI-measure) with PHQ stress evaluations, the number of children at home, and the highest educational level as covariates.

### State Anxiety According to the STAI-S

The assumption on sphericity was significantly violated (*P*<.001) and thus Huynh-Feldt correction was applied (ε=.817). A significant main effect of the PHQ stress evaluation was observed (*P*<.001). Moreover, there was a significant main effect of the group (*P*<.05).

**Figure 5:**
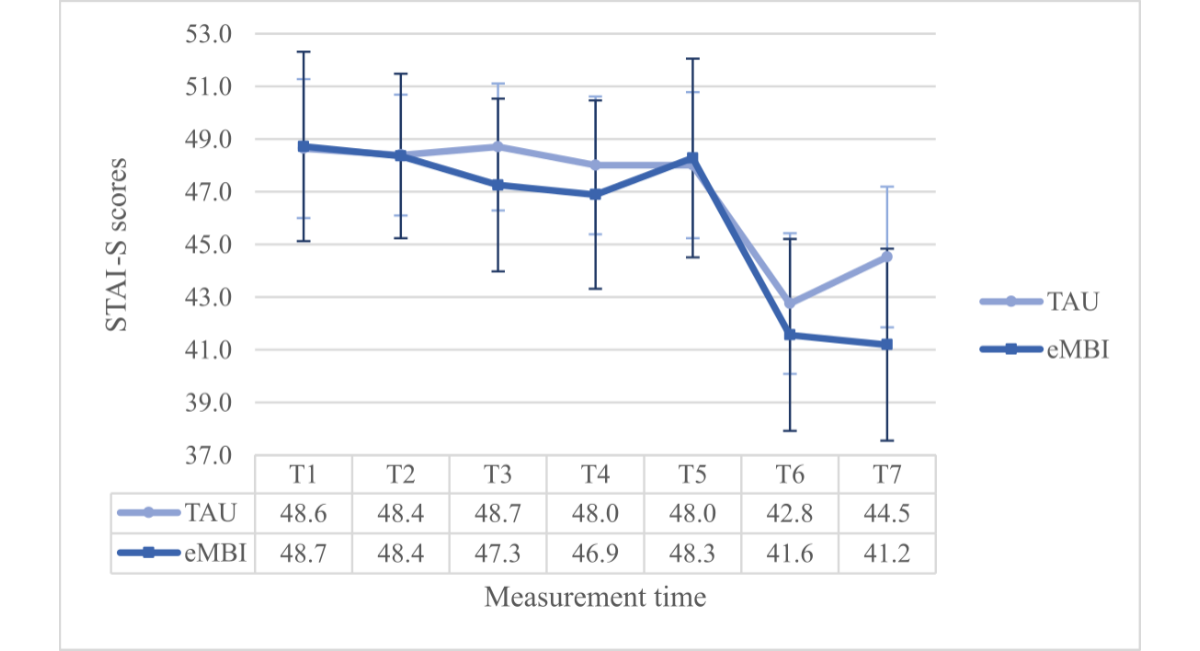
Group x time-interaction effect regarding STAI-S scores for the NPC group. GW: gestational week; PP: postpartum; T1: 28th GW; T2: 30th GW; T3: 32nd GW; T4: 34th GW; T5 = 36th GW; T6: 1 month PP; T7: 5 months PP; TAU: treatment as usual; eMBI: electronic mindfulness-based intervention.

**Figure 6:**
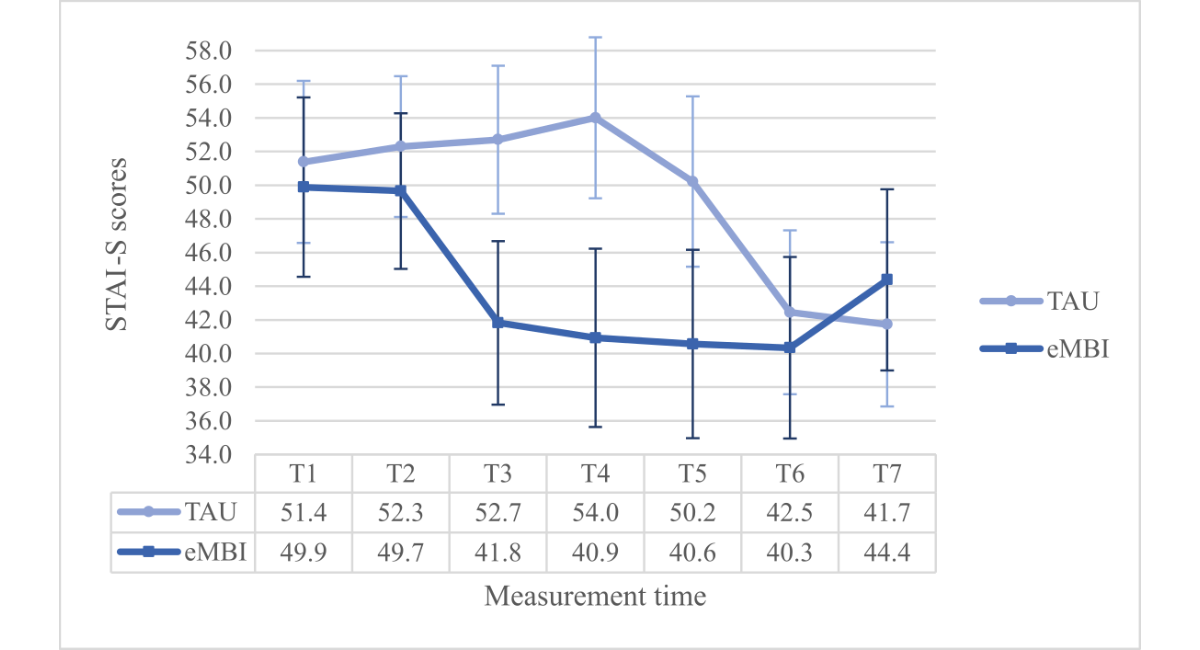
Group x time-interaction effect regarding STAI-S scores for the PC group. eMBI: electronic mindfulness-based intervention; GW: gestational week; PP: postpartum; T1: 28th GW; T2: 30th GW; T3: 32nd GW; T4: 34th GW; T5: 36th GW; T6: 1 month PP; T7: 5 months PP; TAU: treatment as usual.

However, there was a significant interaction effect between group and time (*P*=.01), which was enriched by a significant 3-way interaction term (*F*_4.902,686.330_=4.422, *P*=.001, η²=0.031, ω²=0.016): Dunn’s post hoc test (Ψ_Dunn_=8.8) revealed significant differences between the IG and the CG at T3, T4, and T5, but only in the PC group (Figures 5 and 6).

### Trait Anxiety According to the STAI-T

The assumption on sphericity was significantly violated (*P*<.001) and thus Greenhouse-Geisser correction was applied (ε=.691). A significant main effect of the PHQ stress evaluations was observed (*P*<.001).

**Figure 7:**
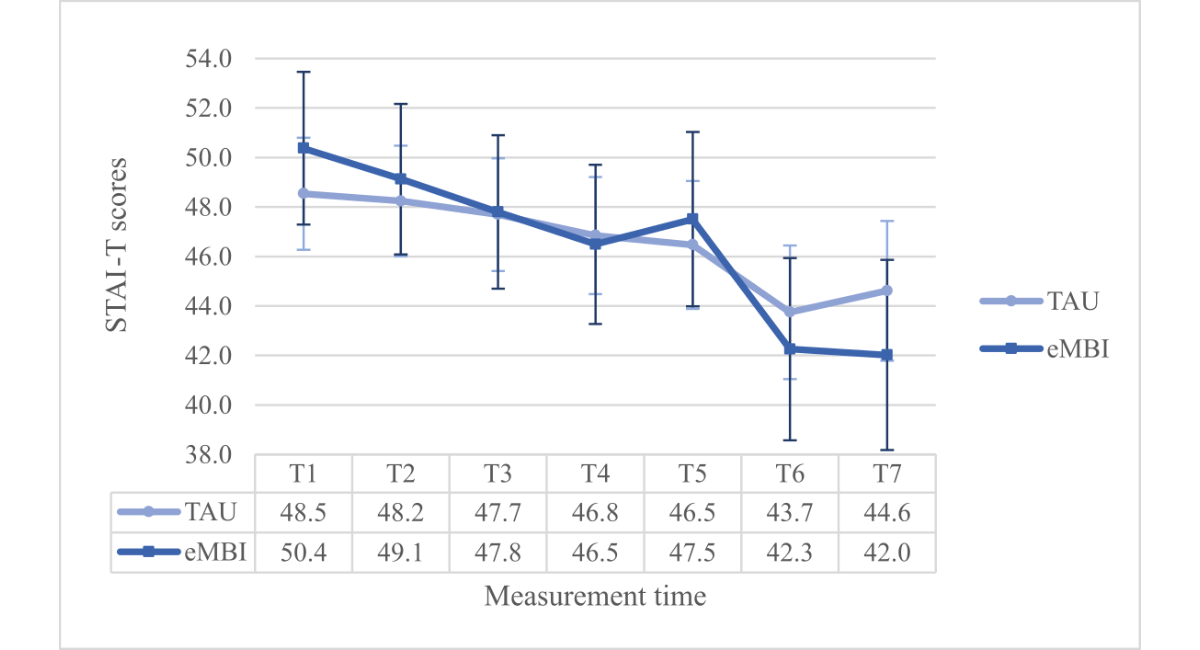
Group x time-interaction effect regarding STAI-T scores for the NPC group. eMBI: electronic mindfulness-based intervention; GW: gestational week; PP: postpartum; T1: 28th GW; T2: 30th GW; T3: 32nd GW; T4: 34th GW; T5: 36th GW; T6: 1 month PP; T7: 5 months PP; TAU: treatment as usual.

**Figure 8:**
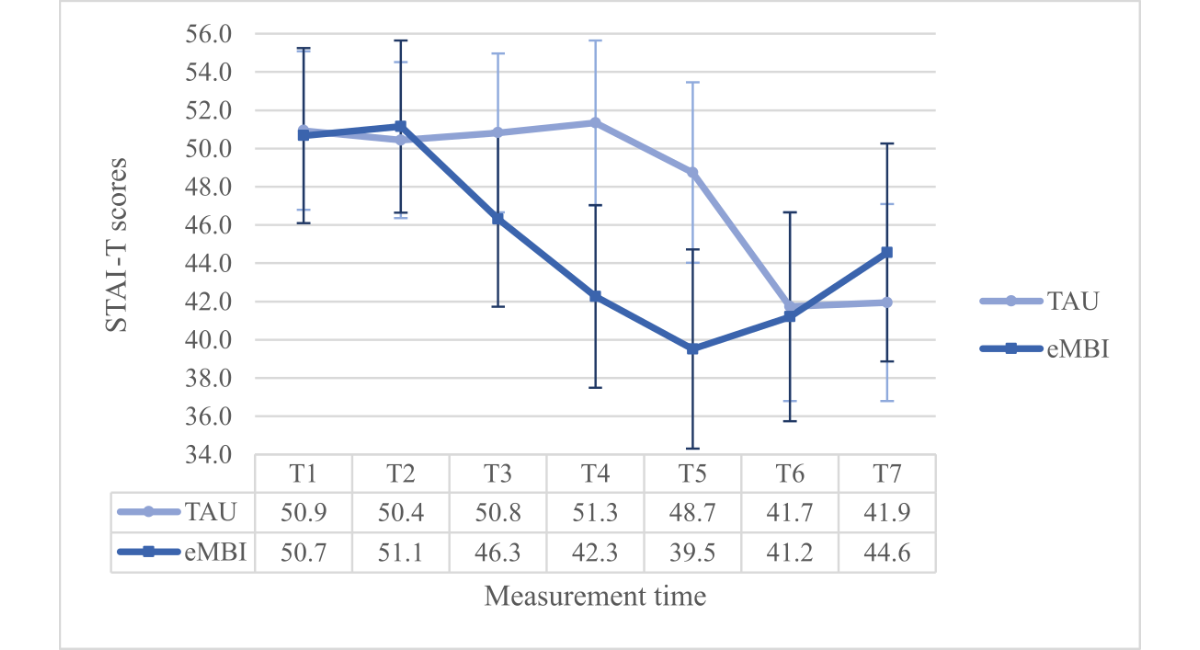
Group x time-interaction effect regarding STAI-T scores for the PC group. eMBI: electronic mindfulness-based intervention; GW: gestational week; PP: postpartum; T1: 28th GW; T2: 30th GW; T3: 32nd GW; T4: 34th GW; T5: 36th GW; T6: 1 month PP; T7: 5 months PP; TAU: treatment as usual.

Moreover, there was a significant interaction effect between group and time (*P*=.02). However, this interaction term was enriched by a significant 3-way interaction term (*F*_4.902,686.330_=4.689, *P*=.001, η²=0.032, ω²=0.015): the Dunn post hoc test (Ψ_Dunn_=8.0) revealed significant differences between the IG and the CG at T4 and T5, but only in the PC group (Figures 7 and 8).

### Pregnancy-Related Anxiety According to the PRAQ-R

As covariates a 2 (group) 2 (coaching) 5 (time) ANOVA with PHQ stress evaluation and number of children were used. Huynh-Feldt correction was applied (ε=.812) as the assumption on sphericity was significantly violated (*P*<.001). We found significant main effects of the PHQ stress evaluation (*P*<.001) and number of children (*P*<.001).

No other significant differences were found between- (*P*≥.10) or within-subject effects (*P*≥.14). In this analysis, the power was additionally virtually 1–β=.998 for small within-subject effects (*f*=0.10). Furthermore, it was 1–β=0.959 for medium-sized between-subject effects (*f*=0.25). Only small between-subject effects cannot be ruled out sufficiently with 1–β=0.264.

### BMI

As a covariate a 2 (group) × 2 (coaching) × 8 (time) – ANOVA with the highest school educational level was used. The assumption on sphericity was significantly violated (*P*<.001) and thus Greenhouse-Geisser correction was applied (ε=.508). There was a significant main effect of time (*P*<.001).

**Figure 9:**
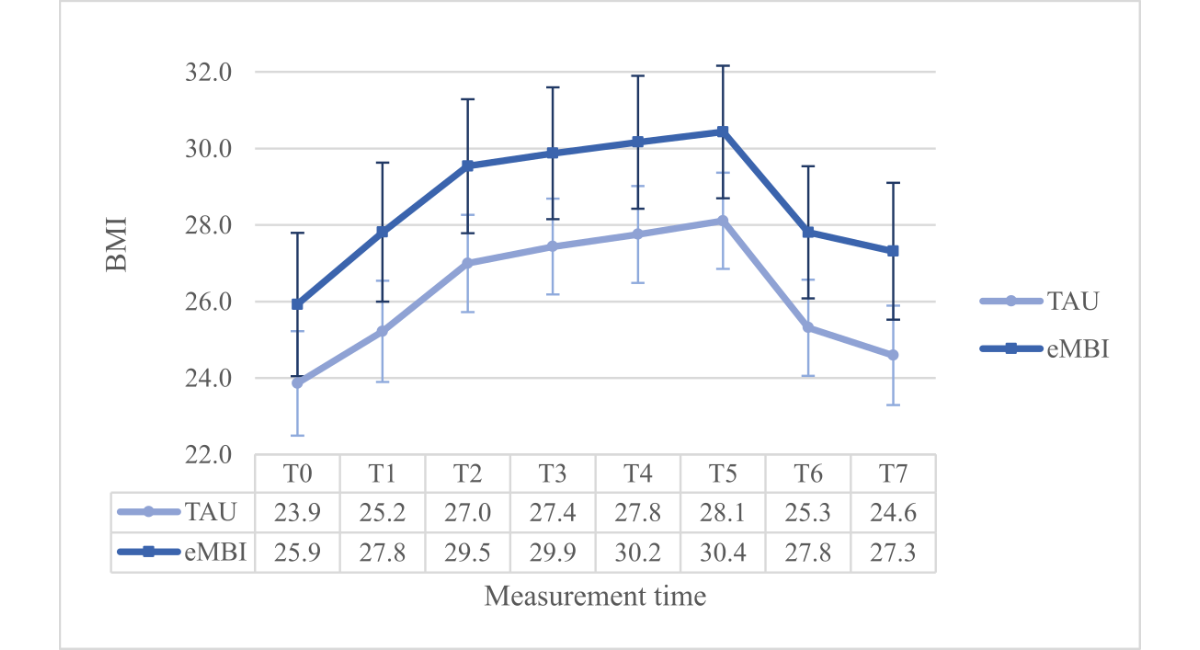
Group x time-interaction effect regarding BMI for the NPC group. eMBI: electronic mindfulness-based intervention; GW: gestational week; PP: postpartum; T0: before pregnancy; T1: 28th GW; T2: 30th GW; T3: 32nd GW; T4: 34th GW; T5: 36th GW; T6: 1 month PP; T7: 5 months PP; TAU: treatment as usual.

**Figure 10:**
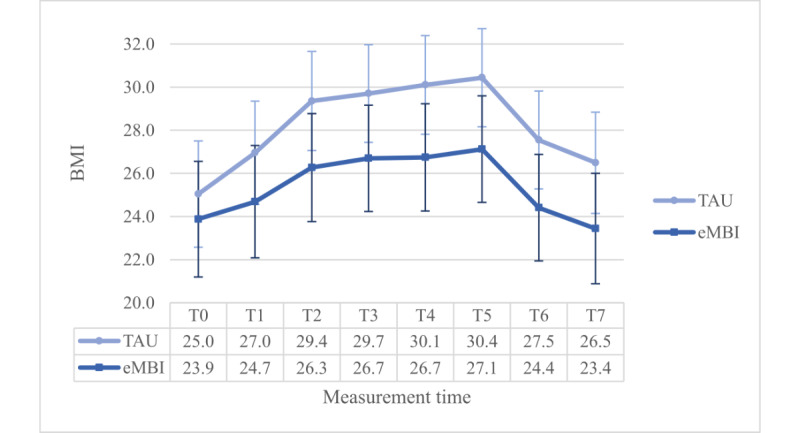
Group x time-interaction effect regarding BMI for the PC group. eMBI: electronic mindfulness-based intervention; GW: gestational week; PP: postpartum; T0: before pregnancy; T1: 28th GW; T2: 30th GW; T3: 32nd GW; T4: 34th GW; T5: 36th GW; T6: 1 month PP; T7: 5 months PP; TAU: treatment as usual.

The group × time--interaction effect was significant, too (*P*=.04). Most importantly, this interaction term was enriched by a significant three-way-interaction term (*F*_3.555, 444.416_=4.732, *P*=.002, η²=0.036, ω²=0.013): the Dunn post hoc test (Ψ_Dunn_=1.5) revealed significant differences at all assessments between the IG and the CG in the NPC group and at all assessments except T0 in the PC group. In the PC group, the IG generally reports a lower BMI than the CG. However, in the NPC group, it is the opposite (Figures 9 and 10).

Moreover, we found a significant main effect of maternal education (*P*=.01). Furthermore, there was a significant interaction effect between the groups with or without coaching (*P*=.01), however, as there was a significant 3-way interaction term as reported above, we did not further investigate this interaction effect.

Findings were nonsignificant for all other between- (*P*≥.57) and within-subject effects (*P*≥.19). In this analysis, the power for even small within-subject effects (*f*=0.10) was virtually 1. The power was 1–β=0.983 for large (*f*=0.40) between-subject effects. Medium-sized (*f*=0.25) and small between-subject effects cannot be ruled out sufficiently with 1–β=.680, 1–β=.142.

## Discussion

### Principal Findings

The aim of the present RCT subanalysis was to examine the effectiveness of a mindfulness-based blended care approach on maternal depression, anxiety, pregnancy- or birth-related anxiety, as well as weight control. Our results show that the eMBI alone has the potential to significantly improve birth- and pregnancy-related anxiety and mindfulness, with long-lasting effects up to 5 months after delivery. However, combining the eMBI with 2.3 PC sessions on average also significantly reduced state and trait anxiety and depression already during pregnancy and, as a side effect, revealed a significant positive influence on maternal BMI. These results favor blended care models combining digital interventions with a minimal amount of personal support by demonstrating the significant effectiveness on perinatal mental health as well as on GWG during pregnancy and the postpartum period. Regarding maternal weight gain, pregnant women in the IG combined with PC generally reported a significantly lower BMI than women in the CG.

### Comparison to Previous Work

The observation that a mindfulness intervention improves the mental health and well-being of pregnant mothers is consistent with findings from previous studies [13,16]. Looking deeper, studies using a mere digital approach often failed to find a benefit of such interventions for psychological distress while face-to-face interventions usually did [41,42]. On the other hand, programs that can be delivered digitally, such as the eMBI program used in our study, appear to be particularly beneficial for a high retention rate [43,44]. Therefore, the results of this subanalysis support the hypothesis that the failure of digital interventions to show effectiveness in lowering depression and general anxiety in contrast to face-to-face interventions might suggest that, in order to positively impact general mental health, a small personal component needs to be added, whereas the positive effects on birth anxiety can be achieved digitally [45]. Therefore, the results of the present study emphasize the importance of personal support in reducing depressive and anxiety symptoms as well as GWG, as these symptoms decreased significantly when participants had 2 or 3 PC sessions on average. In line with this, Bright et al [46] demonstrated the positive effect of interpersonal psychotherapy on maternal well-being during the perinatal period. Favorable findings that support the superiority of a blended care approach compared to a mere digital intervention have been recently published by Martin-Key et al [47] within in the framework of an anonymous online survey in the United Kingdom. Overall, 38% of 829 participating women showed a preference for a blended care approach in order to assess perinatal mental health, whereas only 12% preferred a digital-only consultation [47].

Regarding gestational weight gain and maternal BMI as secondary outcome measures, our results support the hypothesis that digital mindfulness interventions have the potential to positively influence health behaviors including stress and weight management during pregnancy [48]. In particular blended care approaches seem to be effective in providing health-promoting lifestyle modifications and complementing traditional diet and exercise programs [49]. However, in contrast to our study previous ones have not been generated as part of randomized-controlled trials and should be interpreted with caution. Furthermore, differing from previous research, the impact of PC in addition to eMBI on maternal BMI even reached significance in the present sub-analysis of our RCT [48]. However, the number of high-quality and large-scale studies is scarce so far and further research, preferably in the form of an RCT, should be conducted in the future to investigate the feasibility and cost-effectiveness of implementing digital tools into standard clinical routine.

In terms of the respective demographic characteristics results showed that the level of education was significantly associated with the EPDS, STAI-S and STAI-T, and the BMI. According to the current status of the literature former studies find that lower educational levels and socioeconomic status are associated with higher prepregnancy BMI and a higher rate of excessive gestational weight gain [50]. Vice versa, education often correlates not only with socioeconomic status but also with the pursuit of a health-promoting lifestyle [51]. Further negative associations on a significant level have been found between perinatal depression and anxiety on the one hand and educational level on the other hand. The bidirectional association between educational attainment and maternal mental health has been published before [52,53].

Thus, our study results support the use of a blended care approach not only regarding mental health but also in order to promote healthy lifestyle habits and should be taken into account while implementing digital intervention tools into clinical routine. In contrast to previous research, our study results were generated as part of an RCT contributing to good validity and reliability.

### Strengths and Limitations

Our study has the potential to provide high-quality implementation knowledge of a complex mHealth eMBI and coaching intervention for the perinatal period. Being the largest trial across Europe with screening numbers of more than 5000 pregnant women, the Mind: Pregnancy study has the potential to provide high-quality data on the effectiveness of digital eMBI and coaching interventions for preventive and alternative treatment of perinatal mental health problems. Indeed, the results of this evaluation yield important findings that will potentially support the improved health of women and children. The main strength of our study lies in its prospective, longitudinal design with a follow-up period of up to 5 months after childbirth and the inclusion of a CG. The intervention and the peripartum assessments were provided as part of an app and are thus easily and universally applicable and cost-effective. Another strength of our study is that mental health was examined according to a multidimensional approach based on DSM or ICD criteria. In addition, a broad range of confounders were considered.

However, the applicability of the study is limited by several factors that should be considered. First, PC was provided on an as-needed basis during the 8-week intervention period and not in a standardized way, making it difficult to identify a best practice guideline for optimal outcomes. Second, the number of sessions varied, and it remains unclear at what point in the last trimester PC might be most effective in improving mental health. This should be the subject of further research based on the findings of this study. Third, in these analyses, the power to detect in particular small between-subject effects was low. Finally, but not least, the number of observations varies between subgroups. Thus, the results of the interaction terms should be interpreted with caution and should be evaluated in further research. If applicable, a standardized personal component with a fixed amount of sessions should be implemented to improve the validity and reliability of the study findings.

In order to further prove the effect on maternal weight gain, it would be interesting to evaluate the development of the BMI with similar baseline characteristics.

Despite the limitations, our study presents evidence that a blended care approach is potentially highly effective not only in optimizing perinatal mental health but also in affecting maternal weight gain as a synergistic effect. Due to the increasing accessibility of digital interventions, widespread implementation of these interventions should be encouraged.

### Conclusion

In conclusion, our results provide evidence that a blended care model combining digital with a small amount of personal care can potentiate the effect of eMBIs on depressive symptoms and general anxiety with lasting effects up to several months postpartum and can significantly influence maternal BMI. This intervention is a low-cost, easy-to-use intervention for pregnant women to improve their well-being and mental health. However, especially in light of the ongoing digitalization in medical care, it is essential to investigate the potential of individualized, personalized care in everyday clinical practice.

## Appendix

Multimedia Appendix 1 CONSORT-eHEALTH checklist (V 1.6.1).

## Abbreviations

CG: control group
CONSORT: Consolidated Standards of Reporting Trials
DSM-IV: Diagnostic and Statistical Manual of Mental Disorders (Fourth Edition)
eMBI: electronic mindfulness-based interventions
EPDS: Edinburgh Postnatal Depression Scale
EU-GDPR: European General Data Protection Regulation
FFA-14/FMI-14: Freiburg Mindfulness Inventory
GWG: gestational weight gain
IG: intervention group
NPC: no personal coaching
PC: personal coaching
PHQ: Patient Health Questionnaire
PRAQ-R: Pregnancy-Related Anxiety Questionnaire
RCT: randomized controlled trial
SPIRIT: Standard Protocol Items: Recommendations for Intervention Trials
STAI: State-Trait Anxiety Inventory
STAI-S: State-Trait Anxiety Inventory-state scale
STAI-T: State-Trait Anxiety Inventory-trait scale
TAU: treatment as usual
